# Simulation‐free workflow for lattice radiation therapy using deep learning predicted synthetic computed tomography: A feasibility study

**DOI:** 10.1002/acm2.70137

**Published:** 2025-06-12

**Authors:** Libing Zhu, Nathan Y. Yu, Safia K. Ahmed, Jonathan B. Ashman, Diego Santos Toesca, Michael P. Grams, Christopher L. Deufel, Jingwei Duan, Quan Chen, Yi Rong

**Affiliations:** ^1^ Department of Radiation Oncology Mayo Clinic Phoenix Arizona USA; ^2^ Department of Radiation Oncology Mayo Clinic Rochester Minnesota USA; ^3^ Department of Radiation Oncology The University of Alabama at Birmingham Birmingham Alabama USA

**Keywords:** diagnosis CT, expeditious treatment planning, lattice radiation therapy, synthetic CT generation

## Abstract

**Purpose:**

Lattice radiation therapy (LRT) is a form of spatially fractionated radiation therapy that allows increased total dose delivery aiming for improved treatment response without an increase in toxicities, commonly utilized for palliation of bulky tumors. The LRT treatment planning process is complex, while eligible patients often have an urgent need for expedited treatment start. In this study, we aimed to develop a simulation‐free workflow for volumetric modulated arc therapy (VMAT)‐based LRT planning via deep learning‐predicted synthetic CT (sCT) to expedite treatment initiation.

**Methods:**

Two deep learning models were initially trained using 3D U‐Net architecture to generate sCT from diagnostic CTs (dCT) of the thoracic and abdomen regions using a training dataset of 50 patients. The models were then tested on an independent dataset of 15 patients using image similarity analysis assessing *mean absolute error* (MAE) and *structural similarity index measure* (SSIM) as metrics. VMAT‐based LRT plans were generated based on sCT and recalculated on the planning CT (pCT) for dosimetric accuracy comparison. Differences in dose volume histogram (DVH) metrics between pCT and sCT plans were assessed using the Wilcoxon signed‐rank test.

**Results:**

The final sCT prediction model demonstrated high image similarity to pCT, with a MAE and SSIM of 38.93 ± 14.79 Hounsfield Units (HU) and 0.92 ± 0.05 for the thoracic region, and 73.60 ± 22.90 HU and 0.90 ± 0.03 for the abdominal region, respectively. There were no statistically significant differences between sCT and pCT plans in terms of organ‐at‐risk and target volume DVH parameters, including maximum dose (Dmax), mean dose (Dmean), dose delivered to 90% (D90%) and 50% (D50%) of target volume, except for minimum dose (Dmin) and (D10%).

**Conclusion:**

With demonstrated high image similarity and adequate dose agreement between sCT and pCT, our study is a proof‐of‐concept for using deep learning predicted sCT for a simulation‐free treatment planning workflow for VMAT‐based LRT.

## 1 INTRODUCTION

Spatially fractionated radiation therapy (SFRT) is a special form of radiation therapy that purposefully utilizes highly heterogeneous dose plans in which areas of very high dose are intercalated with areas of low dose, allowing the delivery of increased doses to the tumor target without an increase in treatment related toxicities.^1^, ^2^, ^3^, ^4^ SFRT is often used as a “boost dose”, usually immediately preceding the delivery of standard radiation dose regimens, and has demonstrated impressive tumor responses in a variety of malignancies such as gynecologic cancer, gastrointestinal cancer, and radioresistant entities such as osteosarcoma.^5^, ^6^ Literature has shown promising clinical response for patients treated with SFRT.^4^, ^7^ Although not completely understood, the efficacy of SFRT appears to derive from bystander biologic effects in the tumor microenvironment and on patient's immune response caused by the ablative doses intertwined with areas spared from high doses of radiation.^3^

Initial clinical experience with SFRT was based on the utilization of brass blocks termed “GRID block” for partial tumor collimation.^8^, ^9^, ^10^ Although allowing a fast turnaround time between planning and treatment start, GRID blocks are limited by inadequate dose distribution for deep seated tumors and inability to account for tumor motion with respiration. Hence, a volumetric modulated arc therapy (VMAT)‐based SFRT technique was developed named lattice radiation therapy (LRT), which eliminates the necessity of physical blocks.^3^, ^11^ In LRT, a set of spheres are created and distributed in three dimensions inside the gross tumor volume (GTV) while avoiding proximity with sensitive organs‐at‐risk (OARs). A VMAT plan is then optimized to deliver ablative doses to these spheres while limiting dose to the inter‐sphere space, usually with a valley‐to‐peak dose ratio of 0.3–0.4^12^, ^13^ Due to the complex nature of its planning process, LRT utilization is limited when rapid planning turnaround is necessary, common among palliative or symptomatic clinical cases. In a recent prospective Phase I trial on LRT, the median time from simulation until treatment start was 12 days (range: 6–21 days).^14^ Additionally, proton pencil beam scanning (PBS) is being evaluated in an ongoing clinical trial using LRT.^15^

Although efforts in utilizing diagnostic CT (dCT) or magnetic resonance imaging (MRI) for accelerated RT planning workflow have been successful, discrepancies in couch shape, torso or limb positioning, among others, have limited its applicability to all cases.^16^, ^17^, ^18^, ^19^, ^20^ Therefore, dCT‐based treatment planning is mostly used for palliative treatments, which employ less modulated RT plans that are more robust against patient positioning variations.^16^, ^17^, ^18^ MRI‐only based treatment planning with bulk electron density assignment showed promising results for prostate cancer,^19^, ^20^ but is less reliable for lung cancer treatments.^21^ Previous research has demonstrated the feasibility of utilizing deep learning (DL)‐based models to generate synthetic CT (sCT) scans from available dCTs with dosimetric results closely matching those obtained from simulation planning CT (pCT) scans.^22^, ^23^, ^24^, ^25^, ^26^, ^27^, ^28^ Additionally, DL‐based sCT generation has proven viable when utilizing MRI^24^, ^25^ or cone beam CT (CBCT) images.^26^, ^27^

In this study, we explored the feasibility of using DL‐predicted sCT for a simulation‐free LRT treatment planning workflow. First, we developed a DL model to generate sCT scans from available dCTs, to account for couch top differences, and accurately “predict” patients’ simulation pCT features. Secondarily, we sought to validate the dosimetric accuracy of treatment plans created on sCT compared with those based on pCT and estimate the improvement in workflow efficiency by using DL‐predicted sCT plans *in lieu* of simulation pCT plans.

## 2 MATERIALS AND METHODS

### 2.1 Deep learning model development

#### 2.1.1 Dataset identification and pre‐processing

**FIGURE 1:**
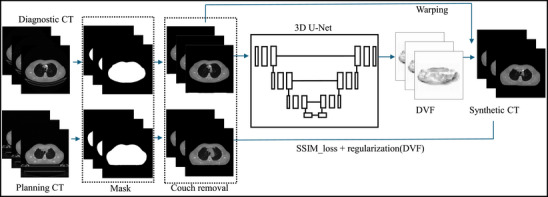
Workflow of synthetic CT prediction using diagnostic CT with couch removed.

After Institutional Review Board (IRB) approval, we retrospectively identified 50 patients for model training and 15 for model testing that were treated at our department and had available diagnostic and simulation planning CT scans. The model testing cohort includes five patients treated with LRT at our clinic and ten cases with sarcoma. To ensure consistency between dCT and pCT, we manually reviewed tumor shape changes. Cases with discrepancies in arm positioning, such as arm‐up in pCT and arm‐down in dCT, were excluded. Additionally, dCT cases with significant truncation were excluded due to incomplete anatomical information. The scanning parameters of dCT is different to that of pCT shown in Table 1. The dCT and pCT scanners share common settings, such as 120 kVp and 500 mm data collection diameter, but differ in other parameters, such as tube current, convolution kernels and filter types, reflecting variations across manufacturers. The mean time interval between dCT and pCT is 12.7 ± 14.04 days, with a range of 1–47 days. The couch in all the CT images was first removed using our custom‐developed algorithm as shown in Figure 1. The process involved thresholding at −200 Hounsfield Units (HU) to segment the patient's body, selecting the largest region in each slice. We then performed a closing operation to fill gaps and applied dilation operation to ensure continuity. The final mask (body: 1, background: 0) was applied to the original CT images, setting the background to −1024 HU for consistency. Both the dCT and pCT images were then resampled to a consistent resolution of 128 × 128 × 64, which were used for training and as the ground truth. Prior to resampling, the dCT was rigidly aligned with the pCT based on the bony structure using MIM software (version 7.2.7, MIM Software Inc., Cleveland, OH, USA) to ensure consistent pixel size and slice thickness.

**TABLE 1:** Scanning parameters of dCT and pCT.

	dCT		pCT
Manufacturer	Philips	GE	Siemens
Convolution kernel	B	–	Br38s
kVp (kV)	120	120	120
Data collection diameter (mm)	500	500	500
x ray tube current (mA)	333	232	177
Exposure (mAs)	77	15	221
Filter type	B	Standard	FLAT
Pixel spacing (mm)	0.9766	0.9766	0.976

Abbreviations: dCT, diagnostic CTs; pCT, planning CT.

#### 2.1.2 Network architecture training

A 3D U‐Net model was trained to predict synthetic CT (sCT) of the thoracic region. The network was implemented in MONAI.^29^ The inputs were dCT images and the ground truth were simulation pCT images as shown in Figure 1. The model's parameters were initialized with a spatial dimension of 3, an input channel of 1, an extraction level of 3, and 32 starting channels. The network architecture featured seven encoding and decoding layers, plus a bottom block, all utilizing 3D convolutional filters, batch normalization, and nonlinear activation functions. The output block produces the deformation vector field (DVF) while preserving the same spatial dimensions as the input. The predicted sCT image was then derived by applying the DVF to the dCT through a warp module.^29^ The loss function consists of image similarity loss^29^ and a regularization term.^23^ Subsequently, the same network architecture was used to train a 3D U‐Net model for predicting sCT in the abdominal region.

#### 2.1.3 Image similarity analysis

Four commonly used metrics, mean absolute error (MAE), normalized cross correlation (NCC), structural similarity index measure (SSIM) and gradient magnitude similarity deviation (GMSD)^30^, ^31^, ^32^ were employed to evaluate the model's performance.^33^ MAE measures the overall HU deviation between sCT and pCT while SSIM assesses structural distortion, luminance and contrast difference, quantifying the similarity between the two CTs. SSIM, calculated with the following equation, ranges from −1 to 1, with 1 indicating perfect image similarity and −1 indicating structural dissimilarities. NCC measures the similarity between two CTs in terms of intensity patterns ranging from −1 to 1, with 1 meaning a perfect correlation. GMSD focuses on the edge preservation across two CTs. The higher the GMSD value, the larger the distortion range, and the lower the image perceptual quality.^30^

(1) $$\mathrm{MAE}=\frac{\sum_{i=1}^{N}\left|X\left(i\right)-Y\left(i\right)\right|}{N}$$

(2) $$\mathrm{NCC}=\frac{\sum_{i=1}^{N}\left(X\left(i\right)-\mu_{x}\right)\left(Y\left(i\right)-\mu_{y}\right)}{\sigma_{x}\sigma_{y}}\;$$

(3) $$\mathrm{SSIM}=\frac{\left(2\mu_{x}\mu_{y}+i\right)\left(2\sigma_{xy}+c2\right)}{\left(\mu_{x}^{2}+\mu_{y}^{2}+c1\right)\left(\sigma_{x}^{2}+\sigma_{y}^{2}+c2\right)}$$

(4) $$\mathrm{GMS}\left(i\right)=\frac{2m_{CT1}\left(i\right)m_{CT2}\left(i\right)+C}{m_{CT1}^{2}\left(i\right)+m_{CT2}^{2}\left(i\right)+C}\;$$

(5) $$\mathrm{GMSD}=\sqrt{\frac{1}{N}\sum_{i=1}^{N}{\left(\mathrm{GMS}\left(i\right)-\bar{\mathrm{GMS}}\right)}^{2}}\;$$

where X(i) and Y(i) are HU values of two CT images at location i, $\mu_{x}$ and $\mu_{y}$ are the mean HU of the two CT images, and $\sigma_{x}$, $\sigma_{y}$ and $\sigma_{xy}$ are the variances of the two images and the covariance between them. c1 and c2 can be found in reference.^31^ GMS(i) is gradient magnitude similarity at location i. $\bar{\mathrm{GMS}}$ is the mean gradient magnitude similarity. $m_{CT1}\left(i\right),$ $\;m_{CT2}\left(i\right)$ are gradient magnitudes at location i after processing with the Prewitt filters. C can be found in the reference.^30^ N is the total number of pixels in the CT.

### 2.2 VMAT‐based LRT treatment planning

Fifteen cases with GTV volumes ranging from 100.7 cc to 1535.9 cc (median = 358.6 cc) were utilized for VMAT‐based LRT treatment planning using the Eclipse treatment planning system (Varian Medical Systems), with a beam model corresponding to the Varian TrueBeam linear accelerator. The target sphere contours were established on sCT by an automated sphere placement algorithm in MATLAB that automatically generates optimized sphere contours with a diameter of 1.5 cm.^34^ The centers of those spheres are within 1.0 cm contraction from the GTV contours, and at least 1.5 cm away from OAR contours. The center‐to‐center distance between spheres in the same axial plane is normally 6–8 cm depending on the case, and no less than 3 cm in the sagittal and coronal planes. Four VMAT fields were utilized with collimator angles of 2°, 358°, 3°, and 357°. The valley doses are typically 30%–40% of prescription between the spheres. The preferred beam energy is 6 MV flattening filter‐free (FFF) due to slightly better dose sparing between spheres. Dose delivered to 50% of the volume (D50%) of each GTV sphere was maintained within ± 50 cGy of the prescription dose of 2000 cGy single fraction. The dose to 0.03 cc (D0.03cc) for all OARs except for skin and the dose to 1cc (D1cc) for skin were kept below 600 cGy. This DVH constraint was chosen considering achievability and safety for LRT and the subsequent contribution to a conventional RT dose.^35^ Detailed optimization steps for VMAT LRT was previously described.^36^ After performing rigid registration between the sCT and clinical pCT based on bony anatomy, the treatment plan optimized on the sCT was recalculated on the pCT for dosimetric comparison (propagating from sCT to pCT). Additionally, contours of pCT were deformably adapted to dCT and the RT plan of sCT was recalculated on the dCT for dosimetric comparison.

### 2.3 Dosimetric evaluation

DVH metrics assessment included the GTV D10%, D50%, D90%, Dmean, Dmin, Dmax, and D50% for all spheres. The metric used for evaluating dose heterogeneity is D10%/D90%. Dmax inside the GTV spheres should typically range from 120% to 150% of the prescription dose. After recalculating 15 cases on pCT, the Wilcoxon signed‐rank test was employed to compute the statistical significance between the DVH values of sCT and pCT. A *p*‐value less than 0.05 was considered statistically significant.

**FIGURE 2:**
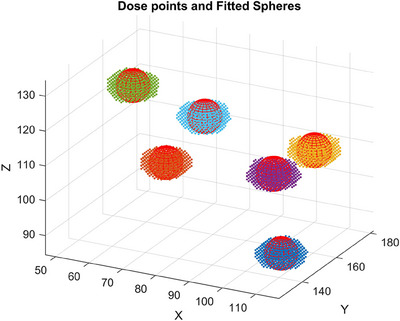
Demonstration of spherical contour creation with diameter 1.5 cm for pCT (50% isodose line segmentation for dose maps shown in dotted points and 1.5 cm diameter sphere creation with the geometric center shown in mesh contours (x, y, z‐axis unit: pixel).

Sphere target contours were created on sCT following clinical standard of procedure protocol, and we developed an algorithm to generate sphere contours on pCT and sCT for dosimetric comparison using spherical fitting. This is to ensure the consistency of sphere contour between pCT and sCT because the spherical target region was created on sCT and there was no spherical target contour on pCT to evaluate the D50% metrics. The algorithm first detected 3D connected regions of the 50% isodose line and then calculated the geometric centroid of the connected regions by determining the mean position of all voxels. A spherical contour with a diameter of 1.5 cm was created at the calculated geometric centroid, shown in Figure 2. The DVH constraint, GTV sphere D50% of all spheres, was computed using the created spherical contours.

## 3 RESULTS

### 3.1 Image similarity assessment

**FIGURE 3:**
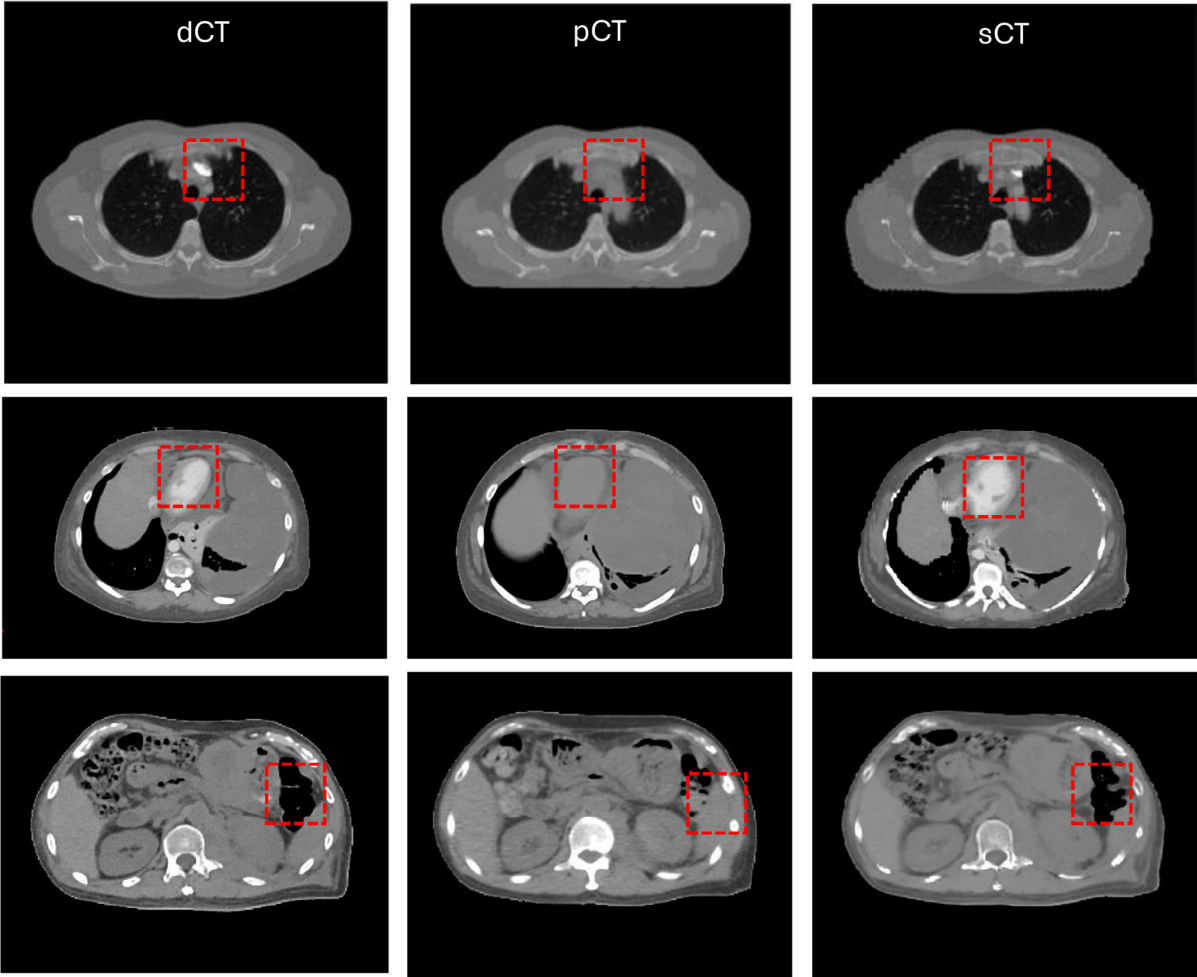
Three example cases of comparison between dCT, pCT, and AI predicted sCT with each row showing one case and dashed red box highlighting the difference.

The performance of the AI‐generated sCT was evaluated against the pCT, with Figure 3 showcasing three significant examples. Notably, the proposed sCT algorithm effectively adapted the patient's body to the flat couch. Furthermore, the lung regions predicted by the AI closely aligned with those in the pCT, demonstrating the model's accuracy in capturing anatomical changes resulting from variations in couch surfaces between dCT and pCT acquisitions. However, some discrepancies were noted in bony, high‐contrast regions and gas‐filled regions, particularly in the stomach due to gastric air, as indicated by the red dashed boxes in the Figure 3.

For the quantitative comparisons between sCT and pCT in the thoracic and abdominal regions, four metrics, MAE, SSIM, NCC and GMSD are shown in Table 2. The MAE and SSIM for sCT versus pCT were 38.9  ± 14.8 HU and 0.92 ± 0.05, respectively, for the thoracic region. The two metrics show worsening values of 73.6 ± 22.9 HU and 0.90 ± 0.03, respectively, in the abdominal region. sCT is more similar to pCT than dCT with obviously improved four metric values. The larger MAE in the abdomen is attributable to the challenging prediction of large and small bowel position due to their inherent physiological mobility. Overall, our results suggest that the DL‐predicted sCT using dCT as input achieves reasonable HU accuracy and structural similarity compared to pCT.

**TABLE 2:** Quantitative comparison results for dCT versus pCT and sCT versus pCT.

	Thorax		Abdomen	
Metrics	dCT versus pCT	sCT versus pCT	dCT versus pCT	sCT versus pCT
MAE	140.69 ± 36.67	38.93 ± 14.79	109.48 ± 22.53	73.60 ± 22.90
SSIM	0.79 ± 0.07	0.92 ± 0.05	0.85 ± 0.04	0.90 ± 0.03
NCC	0.79 ± 0.07	0.92 ± 0.05	0.85 ± 0.04	0.93 ± 0.03
GMSD	0.35 ± 0.02	0.32 ± 0.02	0.34 ± 0.01	0.32 ± 0.01

Abbreviations: dCT, diagnostic CTs; GMSD, gradient magnitude similarity deviation; MAE, mean absolute error; normalized cross correlation;NCC, pCT, planning CT; sCT, synthetic CT; SSIM, structural similarity index measure.

### 3.2 Dosimetric evaluation of sphere targets

**FIGURE 4:**
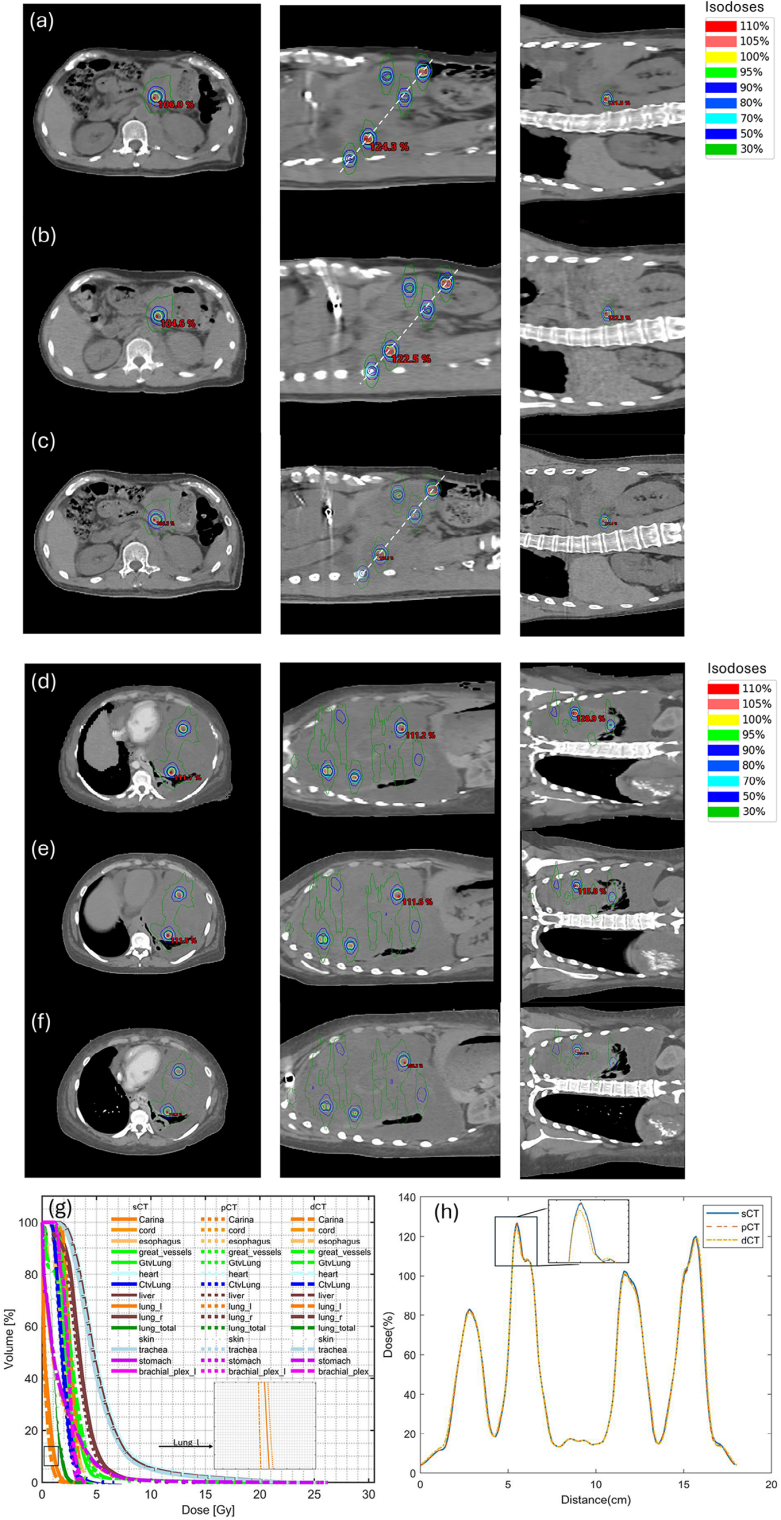
Two examples of dose comparison of LRT on sCT (a, d), recalculated dose on pCT (b, e), and recalculated dose on dCT (c, f) on the axial, coronal and sagittal planes. Panel (g) is the DVH comparison and panel (h) is the dose profile comparison of the white line on panel (a–c) between sCT LRT plan (solid line), pCT LRT plan (dashed line) and dCT LRT plan (dot dash line).

**FIGURE 5:**
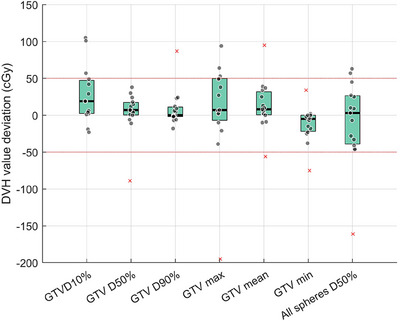
Dose deviations for target DVH parameters between sCT and pCT (outliers that fall outside the whisker of boxplot are shown in cross sign and raw data points that fall within the whisker are shown in dot sign).

Two examples of a VMAT lattice dose distribution of sCT and its recalculation on pCT and dCT are shown in Figures 4a–f. A detailed comparison of dose profiles and DVH curves between sCT, pCT and dCT is demonstrated in Figures 4g–h. Both the dose profile and the DVH curve of sCT are more similar to that of pCT than dCT. As shown in the dose profile comparison (panel h), the sCT‐based LRT is dosimetrically more comparable to the pCT‐based LRT. Fifteen VMAT‐based LRT plans were generated using the sCT, and the dose distribution was compared. The majority of DVH metrics, including GTV D50%, D90%, D10%/D90%, Dmean, Dmax, and all spheres D50% showed no statistically significant difference between sCT and pCT plans, except for GTV D10% and Dmin dose. This implies that the prescription of 20 Gy to the tumor can still be met. The mean absolute dose deviation between sCT and pCT was of 3.6 cGy for GTV D10%, 26.4 cGy for D50%, 8.7 cGy for D90%, 0.006 for D10%/D90% ratio, 8.5 cGy for Dmax, 13.1 cGy for Dmean, 11.7 cGy for Dmin, and 8.3 cGy for D50% of all spheres. Additionally, the 25th and 75th percentiles of all fifteen cases show small deviation within ± 50 cGy, as shown in Figure 5.

### 3.3 Dosimetric evaluation of OARs

**FIGURE 6:**
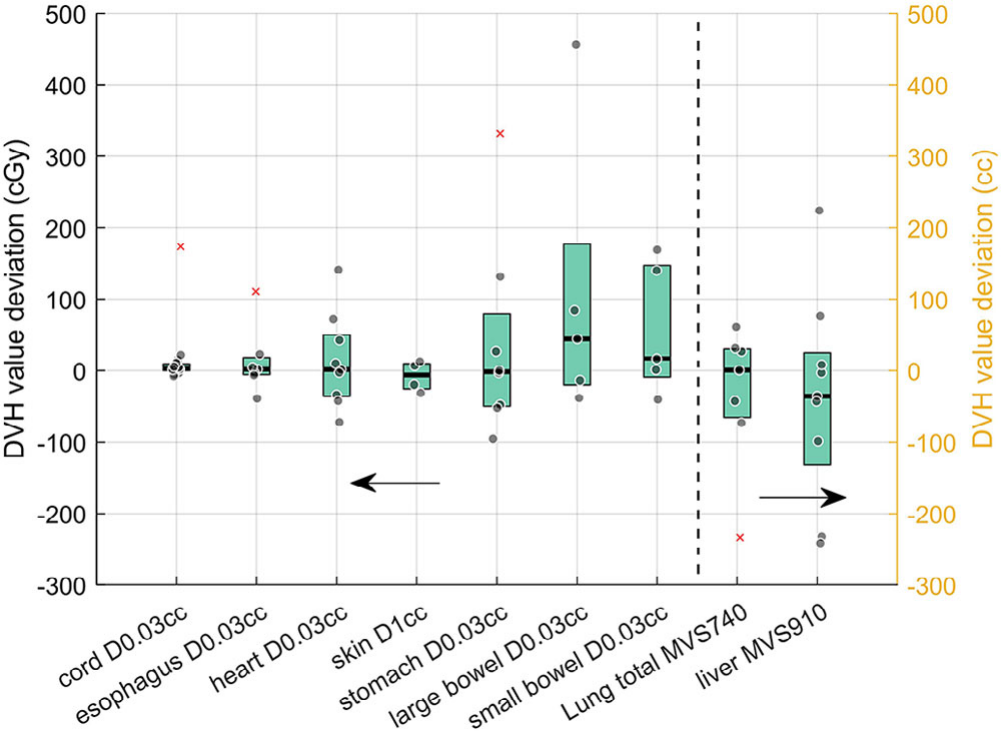
Dose and MVS deviations for OARs DVH parameters between sCT and pCT with lung and liver plotted with the right y axis in the unit of cc (MVS: mean volume sparing, outliers that fall outside the whisker of boxplot are shown in cross sign and raw data points that fall within the whisker are shown in dot sign).

DVH values of sCT and the recalculation on pCT and dCT were compared for the cord, esophagus, heart, skin, total lung, large bowel, small bowel, and liver. The DVH curve of sCT‐based LRT is closer to that of pCT‐based LRT compared with dCT‐based LRT, illustrated in Figure 4g with lung DVH. No statistically significant differences were found for any DVH metrics of all OARs between sCT‐based LRT and pCT‐based LRT, implying good dose accuracy of sCT. After recalculation of treatment plans on pCT, the DVH values of the large bowel and small bowel exceeded the 600 cGy limit, only for one case, due to the anatomical complexity and physiological motion of these two organs. DVH values of the cord, skin, stomach, heart, and esophagus of all cases remain within the 600 cGy constraints (D1cc for skin and D0.03cc for other OARs). Both mean volume sparing (MVS) of the total lung and liver in pCT met the clinical requirements of 1000 cc and 700 cc for the total lung and liver, respectively (lung: MVS740cGy>1000cc, liver: MVS910cGy>700cc). The mean deviation between sCT and pCT was −0.35 cGy for cord D0.03cc, −1.8 cGy for esophagus D0.03cc, 1.5 cGy for heart D0.03cc, 6.3 cGy for skin D1cc, 3.2 cGy for stomach D0.03cc, −44.2 cGy for large bowel D0.03cc, −16.6 cGy for small bowel D0.03cc, −32.61 cc for lung MVS740 cGy, and −30.18 cc for liver MVS910 cGy, as shown in Figure 6.

## 4 DISCUSSION

In this study, we demonstrated the utilization of DL‐predicted sCT creation for the expedited treatment planning of VMAT LRT. The contours and treatment planning are manually performed, which can be further expedited by automated planning.^37^ After acquiring a recent dCT (commonly within a month), the VMAT LRT treatment can be initiated within one day. The sCT‐enabled LRT treatment planning can be initiated without the conventional CT simulation scans. The imaging similarity metrics and dose comparison metrics showed sufficient clinical agreement between the proposed simulation‐free workflow and the conventional workflow. For critically ill patients requiring celerity in treatment initiation, an expedited palliative planning workflow is paramount.

Modern linacs’ onboard capabilities such as cone‐beam CT (CBCT) image‐guided verification or CBCT‐enabled plan adaptation have improved RT delivery accuracy and precision, making the utilization of AI‐based virtual or sCTs a relevant target for time optimization during the RT workflow process.^38^ The MAE for MR‐to‐CT conversions generally falls between 30–80 HU,^25^, ^28^ with high agreement in dosimetric comparisons. In a MR‐sCT synthesis study, the MAE for the head and neck region reached 71.3 HU with a 4‐channel U‐Net and still achieved a high gamma passing rate of 99.0%.^39^ Similarly, a U‐Net study on MR‐sCT synthesis for the abdomen demonstrated an MAE of 105.7 HU and 110.09 HU for the lungs and vertebral bodies respectively. However, the mean deviation across 31 patients was less than 0.15 Gy for targets and OAR DVH metrics.^40^ Another study reported an MAE of 78.7 ± 18.5 HU and 54.3 ± 11.9 HU for the abdomen and pelvis regions, respectively. The two one‐sided test for paired samples (TOST‐P) results implied that all DVH values from sCT for PTV and OARs were equivalent to those from pCT within an equivalence range of ±0.5%.^41^ Additionally, other studies have shown that sCT generation achieved an MAE of 94.1 HU and 89.8 HU with cycle‐consistent generative adversarial networks (cycleGAN) and conditional generative adversarial networks (cGAN), respectively. Both models achieved high dose accuracy with average gamma passing rates higher than 95% (2%/2 mm).^42^ Compared to these MR‐sCT synthesis studies, our study achieved an MAE of 73.6 ± 22.9 HU in the abdomen. This is the first feasibility study demonstrating good performance in image similarity and dose accuracy using DL based sCT for VMAT LRT. D10% and minimum dose in target of sCT show significant difference compared with that of pCT. This is due to that the HU errors in other soft tissue of sCT may significantly affect the delivered dose at target volume. As reported,^43^, ^44^ HU overestimation of sCT can lead to the dose decrease in the target. The outlier where the dose difference in large bowel can be almost 5 Gy can be attributed to the motion of bowels and our AI model cannot predict the bowel motion accurately. This implies that utilization of sCT in gastrointestinal (GI) region is limited due to inaccuracy prediction of bowel motion. Even though the thorax region, especially lungs, can be affected by respiratory motion, the deformation of lungs and heart with AI is more predictable and periodic. The GI region, including bowel and intestines, is highly mobile due to digestion and different filling conditions. This leads to the difficulty for accurate synthetic CT prediction. To avoid potential delivery discrepancies due to large differences between sCT and the actual anatomy, a safeguard mechanism should be implemented for the proposed LRT workflow. During the CBCT image alignment process in a regular LRT workflow, instead of checking target alignment, one important check is to ensure 8 Gy isodose lines are away from the high‐risk organs as shown on the daily CBCT. For the proposed sCT‐based LRT workflow, prior to the treatment delivery, the sCT‐based LRT plan will be recalculated on the daily cone‐beam CT to verify that the radiation delivery is still in the shape of spheres, and the 8 Gy isocenter lines are away from the high‐risk organs.

Our study has a few limitations. First, while the size of our training dataset was adequate for this proof‐of‐concept study, it may not be sufficient for generalized clinical applications. Therefore, evaluating model performance with larger datasets is essential for future applications. We used data augmentation methods^45^ like rotation, translation, and cropping to expand our training dataset. To increase the training data size, each CT scan was rotated by 90 degrees clockwise and cropped with a 128 × 128 window centered on the CT image. Future work will aim to incorporate patient‐specific data into the neural network, such as breath‐hold levels measured with a spirometer.^22^ Second, our model cannot handle CT truncation or differences in arm positioning. Cases with mismatched arm positions between dCT and pCT, or with truncation in the dCT, are excluded due to missing or inconsistent anatomy. Finally, LRT planning followed our institutional protocol. Other LRT protocols should be tested against our model for sCT and pCT dosimetric comparison for external validity.

## 5 CONCLUSION

Our DL model for sCT prediction demonstrated high image similarity to pCT, with significant agreement in LRT DVH metrics. These findings support the feasibility of utilizing sCT to accelerate treatment planning and delivery for VMAT LRT at thoracic region. By streamlining the workflow of palliative RT, this approach has the potential to reduce patient waiting time for treatment start and provide symptom relief in a timelier manner. The proposed workflow can be generalizable to palliative cases that require a fast turnaround from diagnosis to radiotherapy treatments. Further studies are underway to test this workflow for those cases.

## AUTHOR CONTRIBUTIONS

*Conceptualization*: Libing Zhu, Nathan Y. Yu, Yi Rong. *Data curation*: Libing Zhu,Safia K. Ahmed. *Formal analysis*: Libing Zhu, Safia K. Ahmed. *Investigation*: Libing Zhu, Jonathan B. Ashman, Diego Santos Toesca. *Methodology*: Libing Zhu, Safia K. Ahmed, Nathan Y. Yu, Jonathan B. Ashman, Diego Santos Toesca, Michael P. Grams, Chris L. Deufel, Jingwei Duan, Quan Chen, Yi Rong. *Project administration*: Yi Rong. *Resources*: Michael P. Grams. *Software*: Libing Zhu. *Supervision*: Quan Chen, Yi Rong. *Validation*: Libing Zhu, Nathan Y. Yu, Quan Chen, Yi Rong. *Visualization*: Libing Zhu. *Writing–review & editing*: Libing Zhu, Nathan Y. Yu, Safia K. Ahmed, Jonathan B. Ashman, Diego Santos Toesca, Michael P. Grams, Chris L. Deufel, Jingwei Duan, Quan Chen, Yi Rong. *Writing–original draft*: Libing Zhu.

## CONFLICT OF INTEREST STATEMENT

Quan Chen is a co‐founder of Carina Medical LLC. All other authors have no conflict of interest to declare.

## INSTITUTIONAL REVIEW BOARD STATEMENT

The study was conducted in accordance with the Declaration of Helsinki and approved by the Institutional Review Board (or Ethics Committee) of Mayo Clinic (protocol code 23‐007483 and date of approval: Aug 29th, 2023).

## Data Availability

Original data will be shared upon request to the corresponding author.
